# Molecular detection, isolation and characterization of Peste‐des‐petits ruminants virus from goat milk from outbreaks in Bangladesh and its implication for eradication strategy

**DOI:** 10.1111/tbed.12911

**Published:** 2018-05-28

**Authors:** Brian Donald Clarke, Mohammad Rafiqul Islam, Mohammad Abu Yusuf, Mana Mahapatra, Satya Parida

**Affiliations:** ^1^ The Pirbright Institute Woking UK; ^2^ Bangladesh Agricultural Research Council Farmgate Bangladesh; ^3^ SAARC Regional Leading Diagnostic Laboratory for PPR Bangladesh Livestock Research Institute Savar Bangladesh

**Keywords:** goats, lineage IV PPRV, morbillivirus, noninvasive sample, phylogenetic analysis, PPR, PPR virus in milk

## Abstract

*Peste‐des‐petits ruminants* (PPR) is a highly contagious transboundary viral disease of small ruminants, which is endemic in much of Africa, the Middle East and Asia. In South Asia, PPR is of significant concern to the Indian subcontinent including Bangladesh as more than 30% of the world's sheep and goats are farmed in this region, predominantly by small, poor and marginal farmers. PPR virus was detected and isolated from goat milk from field samples from PPR outbreaks (2012–2015) in Bangladesh and its full‐length sequences obtained. Sequence analysis of the partial N gene of Bangladesh isolates showed 99.3%–100% identity whereas 98.2%–99.6% identity was observed when compared with neighbouring Indian viruses. Further analysis of the full‐length genomes indicated that the Bangladesh isolates were 99.3%–99.99% identical among themselves and 98.3%–98.4% identical to neighbouring Indian viruses. These findings further support the transboundary transmission of PPR virus across the Indian and Bangladesh border. In additional, the establishment of a cross‐border strategy between India and Bangladesh will be of paramount importance for the eradication of PPR in this region. Molecular detection and isolation of PPR virus from milk is of significant potential concern for spread of the disease to free areas as the major producers of goat milk globally are PPR endemic countries in particular India and Bangladesh, as well as Sudan. Milk is a noninvasive sample type and bulk goat milk sampling for the detection of PPRV would be of practical significance for regional surveillance of PPRV as progress is made towards the targeted 2030 eradication.

## 1 INTRODUCTION

*Peste‐des‐petits ruminants* (PPR) is the most important OIE listed disease of small farmed ruminants in the developing world (Baron, Parida, & Oura, 2011; Parida et al., 2016). The etiological agent, PPR virus (PPRV) is a member of the family *Paramyxoviridae* and genus *Morbillivirus* (Banyard et al., 2010). Following the eradication of rinderpest, PPRV has been identified by the Food and Agriculture Organisation (FAO) and World Organisation for Animal Health (OIE) as the next target for eradication by the year 2030. PPRV exists as single serotype, which groups into four distinct lineages (I–IV) based on sequence comparison of the C‐terminus of the N gene (Couacy‐Hymann et al., 2002) and F gene (Forsyth & Barrett, 1995). PPR was first identified in Cote d'Ivoire (Ivory Coast) in 1942 as an entity distinct from rinderpest (Gargadennec & Lalanne, 1942). With the notable exception of most southern African countries (South Africa, Botswana, Namibia, Zimbabwe, Mozambique and Malawi), it is now recognized to be endemic throughout Africa as well as the Middle East, Central, East and south Asia. Lineage IV is the primary circulating lineage of PPRV in the Middle East and Asia, with recent incursions into China and Tibet (Banyard, Wang, & Parida, 2014; Wang et al., 2009) into North (Baazizi et al., 2017; Fakri et al., 2016; Muniraju et al., 2013), Central (Maganga et al., 2013), and East Africa as far south as Tanzania (Lembo et al., 2013; Mahapatra et al., 2015).

Lineage IV PPRV was first confirmed in the Indian Subcontinent in North India in 1994 although reports of single outbreak of Lineage III PPR in India date back to 1987 (Nanda et al., 1996; Shaila, Purushothaman, Bhavasar, Venugopal, & Venkatesan, 1989) and has subsequently been reported in the neighbouring Pakistan in 1994 (Amjad, Qamar Ul, Forsyth, Barrett, & Rossiter, 1996), Bangladesh (Islam, Shamsuddin, Das, & Dewan, 2001) in 1993, Nepal (Dhar et al., 2002) in 1995, and Bhutan (Parida et al., 2015). Within Bangladesh, PPR is considered endemic since 1993 (Islam et al., 2001), and the mean morbidity and mortality rates are of approximately 79% and 59%, respectively whereas seropositivity is seen in the range of 20%–30% (Bhuiyan, 2012; Rony, Rahman, Alam, Dhand, & Ward, 2017).

Bangladesh is home to the 5th largest population of goats with more than 55 million animals estimated by the FAO in 2014, behind China, India, Nigeria and Pakistan, and has the largest population by land mass (FAO, 2016). The overwhelming majority of these animals are raised in small holdings by poor, marginal and subsistence farmers (Sarker & Islam, 2011). Goat meat makes up greater than 38% of the total meat production in Bangladesh and more than 11% of the goat milk produced globally is produced within Bangladesh (FAO, 2016), and greater than 55% of the milk consumed annually in Bangladesh is from goats, as such the containment of PPRV is of significant concern to the region.

This publication describes for the first time the molecular detection as well as isolation and molecular characterization of full‐length PPRV from goat milk (noninvasive sample). Further, Bayesian analysis of full‐length PPRV genomes and neighbourhood‐joining phylogenetic analysis of partial N gene of PPRV from various outbreaks in Bangladesh between 2012 and 2015 were included in the study.

## 2 MATERIALS AND METHODS

### 2.1 Sample collection

**Figure 1:**
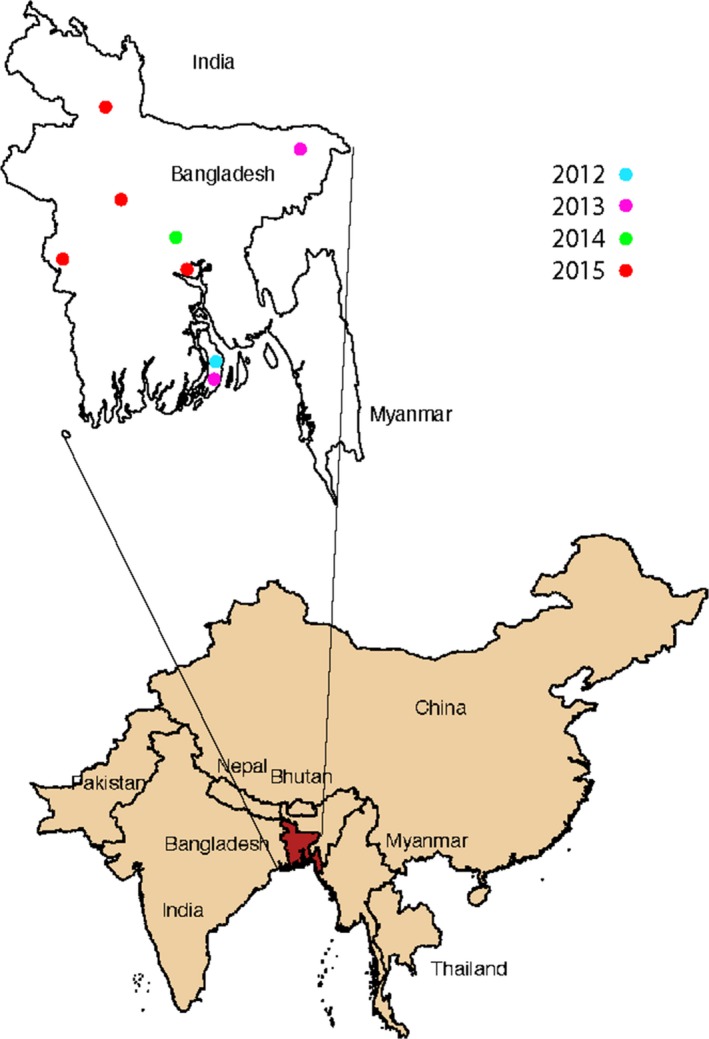
Locations of sampled PPR outbreaks in Bangladesh. Inset: Bangladesh (Red) and surrounding transboundary region (Tan)

Samples were collected across a 3‐year period from 8 locations (Figure 1) as part of routine diagnostic procedures for PPR in Bangladesh (Table 1). Samples included nasal swabs, tissue samples predominantly from lung, as well as milk and faecal samples. Selected samples (*n* = 19) were shipped on dry ice to The Pirbright Institute for confirmation of diagnosis and molecular testing.

**Table 1:** Details of the samples employed in this study

Serial No.	Sample identification No.	Date of collection	Place of collection	Sample type
1	170	30.06.2012	Bhola	Lung
2	167	2.02.2013	Sylhet	Nasal Swab
3	174	17.11.2013	Bhola	Lung
4	147	05.03.2014	Gangi, Meherpur	Nasal Swab
5	70	14.05.2015	Sirajgonj	Nasal Swab
6	53	09.06.2015	Savar	Milk
7	54	09.06.2015	Savar	Lung
8	40	12.06.2015	Chuadanga	Nasal Swab
9	51	12.06.2015	Chuadanga	Faeces
10	52	12.06.2015	Chuadanga	Milk
11	27	04.07.2015	Munsigonj	Nasal Swab
12	2	13.07.2015	Nihkanchari	Nasal Swab
13‐14	18‐19	13.07.2015	Nihkanchari	Milk
15‐19	20‐24	13.07.2015	Nihkanchari	Faeces

### 2.2 Ethics statement

As samples were collected for the diagnosis of PPR during the course of usual veterinary diagnostic procedures in Bangladesh, no permits were required for collection. The samples were sent to the Pirbright Institute (hosts the PPR reference laboratory) for further diagnosis and molecular characterization. Upon consultation, the local Pirbright animal welfare ethical review board (AWERB) confirmed that no requirements for additional approvals were needed as the samples were collected primarily for veterinary diagnostic purposes in Bangladesh and not for the direct purposes of research. Tissue samples were collected from dead animals only.

### 2.3 Virus isolation

Attempts were made to isolate virus from tissue samples, nasal swabs, as well as faecal material and from goat milk. The tissue samples and nasal swabs were processed as described previously (Clarke et al., 2017). For faecal material, where solid pellets were present approximately 1 g of faecal matter was homogenized in 3 ml of M25 buffer using a mortar and pestle; from diarrhoea faecal samples approximately 1 ml of material was diluted into 3 ml of M25 supplemented as above and any solid fragments triturated with mortar and pestle. Milk samples were diluted 1:10 in PBS with antibiotics as above. Homogenates were clarified by centrifugation at 1000 × *g* for 15 minutes at +4°C and 500 μl of the supernatant was inoculated onto 70% confluent Vero dog slam cells (VDS) and incubated for 2 hr at 37°C in an atmosphere of 5% CO_2_, before the inoculant was replaced with 5 ml of Dulbecco's Modified Eagle's medium (DMEM) supplemented with 5% foetal calf serum (FCS). The cells were incubated for up to 7 days and blindly passaged or until cytopathic effects (CPE) were observed. The samples were passaged at least five times before declaring negative.

### 2.4 RNA extraction, reverse transcription (RT), polymerase chain reaction (PCR), real‐time RT‐PCR (qRT‐PCR) and sequencing

Total RNA was extracted from the homogenized tissue samples, faecal matter, nasal swabs and milk samples using Trizol™ (Invitrogen) as per the manufacturer's instructions. In addition, the eluted RNA from faecal samples was further purified using the RNeasy mini RNA Extraction Kit (Qiagen) to remove PCR inhibitors present in faecal material following the manufacturer's protocol after dilution to 100 μl in nuclease‐free water. RT‐PCR to amplify the C‐terminus of the N gene was carried out as previously described (Baazizi et al., 2017). In additional, milk samples were analyzed by qRT‐PCR to assess the viral load (Batten et al., 2011) using Superscript III Platinum R one step qRT‐PCR system kit (Invitrogen).

For full‐length genome sequencing a hemi‐nested RT‐PCR was performed on tissue samples as described previously (Muniraju, Munir, Banyard et al., 2014) and amplification of the terminal 5′ and 3′ ends of the PPRV genome was accomplished via RACE, as previously described (Bao et al., 2012; Muniraju, Munir, Banyard et al., 2014). The PCR amplicons were purified and sequenced as previously described (Clarke et al., 2017).

### 2.5 Sequence analysis

Both partial N and full‐length sequences were assembled and analyzed using SeqMan pro (DNAStar Lasergene 13.0). Nucleotide sequences of the viruses were aligned using the CLUSTAL X multiple sequence alignment programme (Thompson, Gibson, & Higgins, 2002) or MUSCLE as appropriate (Edgar, 2004).

For sequence data not generated in this study complete PPRV genome sequences (Supporting information Table S1; *n* = 37) were obtained from GenBank (as on 15/12/2017). Sequences obtained from live attenuated vaccine virus strains (India/Sungri 96: KJ867542, KF727981 and Nigeria 75/1 (X74443, HQ197753) were removed prior to analysis, these sequences have previously been shown to substantially skew phylogenetic analyses (Muniraju, Munir, Parthiban et al., 2014). To identify the nearest common ancestor and hence likely dates of divergence, the Bangladesh sequence was compared using the coalescent‐based Bayesian Markov chain Monte Carlo (MCMC) (Drummond & Rambaut, 2007; Drummond, Suchard, Xie, & Rambaut, 2012) approach to all available full‐length PPRV wild‐type genomes available (Supporting information Table S1). The general time‐reversible nucleotide substitution model with gamma distribution and invariant sites was selected on the basis of Bayes factor results following path sampling (data not shown). Path sampling was performed until the marginal likelihood estimate remained constant (Nr = 16). As has been previously determined the relaxed uncorrelated exponential distribution (UCED) clock model (Drummond, Ho, Phillips, & Rambaut, 2006) was the best fit to PPRV complete genomes (Muniraju, Munir, Parthiban et al., 2014; Parida et al., 2015). As there are very few (*n* = 4) (KR261605, KT270355, KR140086, and KX033350) full‐length sequences available for the surrounding region, further phylogenic analysis was undertaken using the PPRV partial N gene sequence data of the C‐terminal region of the N gene.

Partial N sequences to be included in the analysis were selected from GenBank on the basis of accurate annotations including locations and dates of sampling as well as uniqueness. Sequences which had identical nucleotides (genome position 1360–1614, 255 nt), year and location were discarded leaving a final dataset of 171 partial N sequences (Supporting information Table S2) to which the 13 sequences generated in this study were added, making it 184 in total. The partial N dataset was aligned using MUSCLE and phylogenetic analyses were performed using MEGA6 (Tamura, Stecher, Peterson, Filipski, & Kumar, 2013). The neighbour‐joining tree was generated using the Kimura 2‐parameter model and tests for phylogeny performed using the bootstrap method with 10,000 replications and the gaps/missing data removed by pairwise deletion (Kimura, 1980).

## 3 RESULTS AND DISCUSSION

### 3.1 Genome detection, virus isolation from PPR infected goat milk and its implication

All samples were tested for PPRV using primers targeting the highly variable C‐terminus of the N gene followed by hemi‐nested PCR using the same primer pair if initial PCR was negative (Table 2). The faecal samples were found to be weak positive in PCR. Milk sample analysis by real‐time RT‐PCR revealed high viral load (Table 2).

**Table 2:** PCR and virus isolation results

Sample name	Sample type	Virus isolated	PCR/qRT‐PCR^a^	Nested PCR	Partial N sequence	Full‐length sequence
Bangladesh/B170/Bhola/2012	Lung	+	+		+	
Bangladesh/B167/Sylhet/2013	Nasal Swab		+		+	
Bangladesh/B174/Bhola/2013	Lung	+	+		+	
Bangladesh/B147/Gangi/2013	Nasal Swab		+		+	
Bangladesh/B70/Sirajgonj/2014	Nasal Swab		+		+	
Bangladesh/B53/Savar/2015	Milk	+	+/30.1		+	
Bangladesh/B54/Savar/2015	Lung		+		+	
Bangladesh/B40/Chuadanga/2015	Nasal Swab		+		+	
Bangladesh/B51/Chuadanga/2015	Faeces		+		+	
Bangladesh/B52/Chuadanga/2015	Milk		+/24.76		+	
Bangladesh/B27/Munsigonj/2015	Nasal Swab		+			+
Bangladesh/B2/Nihkanchari/2015	Nasal Swab		+			+
Bangladesh/B18/Nihkanchari/2015	Milk	+	+/24.61		+	
Bangladesh/B19/Nihkanchari/2015	Milk	+	+/23.54			+
Bangladesh/B20/Nihkanchari/2015	Faeces		+			
Bangladesh/B21/Nihkanchari/2015	Faeces					
Bangladesh/B22/Nihkanchari/2015	Faeces		+			
Bangladesh/B23/Nihkanchari/2015	Faeces			+		
Bangladesh/B24/Nihkanchari/2015	Faeces					

*Note*. The qRT‐PCR results are presented as cycle threshold (Ct) values where applicable. The empty cells indicate negative result. ^a^qRT‐PCR was carried out only on milk samples.

At present, there are limited data (Wasee Ullah et al., 2016) regarding the successful isolation of infectious virus from faecal material and no attempts are currently documented as to the isolation of virus from milk. Therefore, samples which were positive in PCR were inoculated onto VDS cells and passaged. Virus was successfully isolated from a total of three milk samples (Table 2). Of these two samples, Bangladesh/B18/Nihkanchari/2015 and Bangladesh/B19/Nihkanchari/2015 were collected from the same region and sampled on the same day. Bangladesh/B19/Nihkanchari/2015 and Bangladesh/B53/Savar/2015 showed obvious CPE including large syncytia and cell fusion 3 days post‐inoculation, CPE was not observed in Bangladesh/B18/Nihkanchari/2015 until day 4 following three blind passages. No PPRV specific CPE was observed following any passage from any faecal sample, some nonspecific toxicity was observed following initial inoculation of faecal homogenates, however, no effect was observed following subsequent blind passages. Virus was additionally isolated from lung tissues from two samples Bangladesh/B170/Bhola/2012 and Bangladesh/B174/Bhola/2013.

The isolation of infectious virus from milk has implications not just for the vertical transmission of PPR within animal herds but also for the spread of PPRV within endemic regions and across regional boundaries and borders due to export and import of infected milk. India (30%), Sudan (17%) and importantly for this study Bangladesh (11%) are the largest global producers of goat milk (Pacinovski et al., 2015; Wijesinha‐Bettoni, Burlingame, Muehlhoff, Bennett, & McMahon, 2013) and each is considered endemic for PPR. Whilst raw goat milk and unpasteurized products as well as other sheep and goat products are highly restricted imports into Europe (EU regulation 1308/2013) and other similarly developed counties (US FDA regulation MI‐00‐4), similar restrictions do not exist, or are routinely disregarded for cultural and practical reasons in PPR endemic regions. Within Bangladesh, goat milk is predominantly produced by small‐holders and shipped to regional co‐operative processing plants (Hemme, Garcia, & Khan, 2004). This movement of milk and the associated equipment and personal is a possible source of fomites as has been observed for foot‐and‐mouth disease virus (FMDV) (Donaldson, 1997; Reid et al., 2006). However, these larger milk processing facilities also provide an ideal location for testing for the presence of PPRV genome within the region and the establishment of a robust calibrated test either via conventional or quantitative PCR should be prioritized for bulk milk samples. Although the number of samples tested in this study is relatively low, the load of PPR virus genome detected in goat milk was similar to that of FMDV as reported by Reid et al. (2006). Bulk milk testing has been proposed for surveillance of FMDV (Reid et al., 2006) and the development of equivalent tests for PPRV would be of significant utility for regional surveillance of PPRV as progress is made towards the 2030 eradication of PPRV. Further for diagnosis of PPR invasive sample types such as nasal, mouth and eye swabs, and blood samples are usually collected by veterinarians from sick animals that may cause stress to the animal whereas milk sample will serve as a noninvasive method, a much‐preferred method of sample collection.

### 3.2 Molecular characterization of PPRV isolated from milk, faecal samples and tissues

To determine the effect of multiple passages on PPRV, full‐length sequencing was performed on Bangladesh/B19/Nihkanchari/2015 prior to passaging and following three rounds of passage. A single nucleotide shift A–G was observed at position 9,203 in the noncoding region between the hemagglutinin (H) and viral polymerase (L) genes. This result confirms previous work (Wu et al., 2016) that small passage numbers of PPRV result few if any changes in the genome and additionally that care should be taken that the whole genome of PPRV, not simply the coding regions are sequenced as previous published data following serial passaging has focused upon the coding regions (Wu et al., 2016).

**Figure 2:**
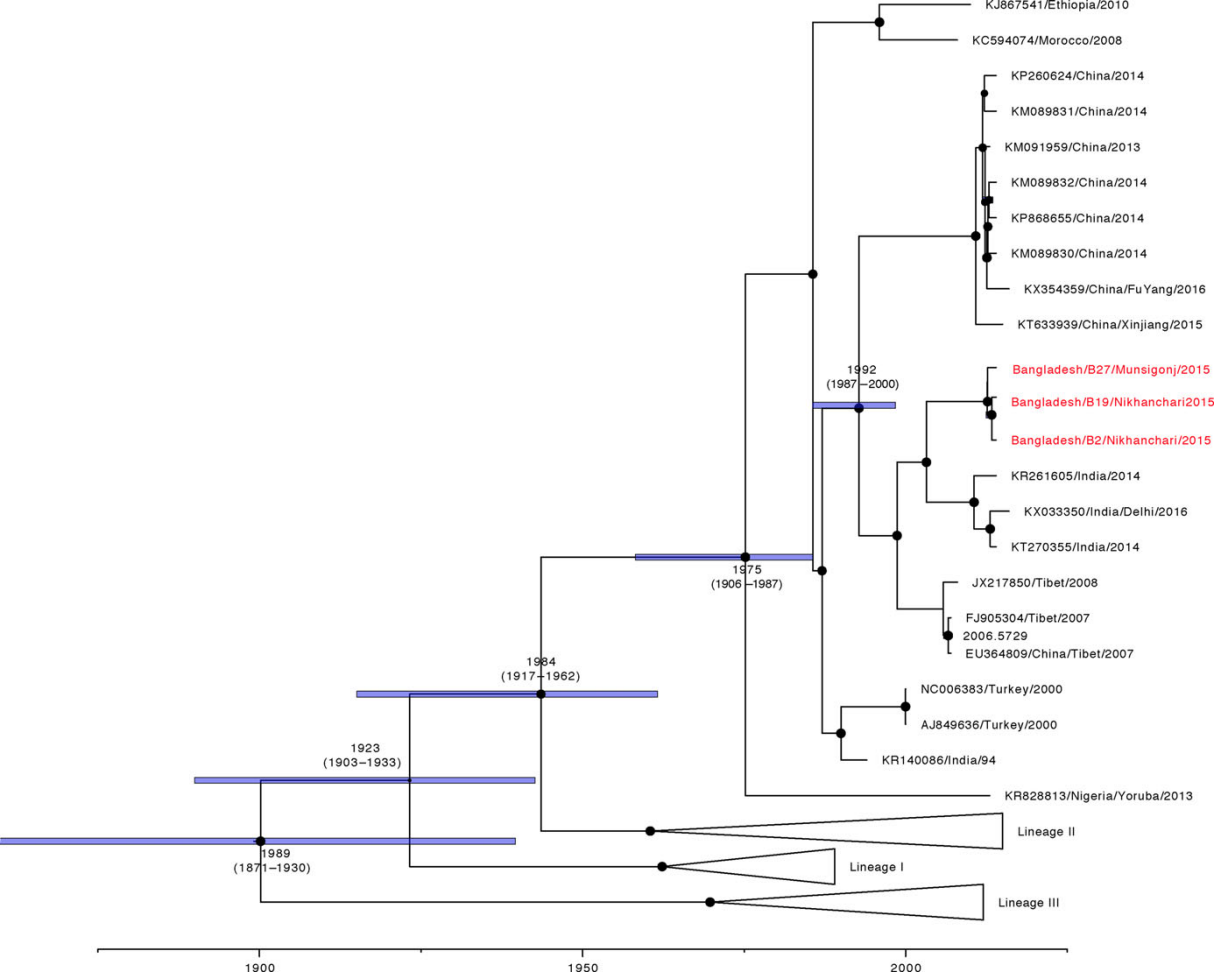
Maximum clade credibility (MCC) tree from Bayesian analysis of full‐length PPRV genomes. The posterior probabilities are indicated by the size of the node, and TMRCA and 95% HPD of the branches are depicted. Accession number, country of origin and sampling year of each isolate is shown. All full‐length sequences generated in this study are highlighted in red and have been submitted to NCBI and awaiting accession numbers

Full‐length sequencing of PPRV isolated from lung tissues from Nihkanchari (Bangladesh/B2/Nihkanchari/2015), Munsigonj (Bangladesh/B27/Munsigonj/2015) as well as from the milk sample (Bangladesh/B19/Nihkanchari/2015) was performed. As expected, all genomes were 15,948 nucleotide long, and the genomic structure of the genomes was also as expected. Samples from Nihkanchari (Bangladesh/B2/Nihkanchari/2015 and Bangladesh/B19/Nihkanchari/2015) were 99.83% identical, and the sample from Munsigonj (Bangladesh/B27/Munsigonj/2015) 99.36% identical to both the samples. All isolates grouped within lineage IV viruses as expected. These viruses were most closely related (>98% identical) to sequences from Tamil Nadu (2014) and Delhi (2016) in India (KT270355/India/Tamil_Nadu/2014‐ 98.1%, KR261605/India/Tamil_Nadu/2014‐ 98.2%, KX033350/India/Delhi/2016‐ 98.2%) and from Tibet (JX217850/Tibet/2008‐ 98.2%, FJ905304/Tibet/2007‐ 98.3%, EU364809/China/Tibet/2007‐ 98.2%). To further compare the Bangladesh full‐length sequences, a Bayesian time‐scaled maximum clade credibility (MCC) maximum likelihood tree was constructed including the full length Bangladesh/B2/Nihkanchari/2015, Bangladesh/B19/Nihkanchari/2015 and Bangladesh/B27/Munsigonj/2015 as well as all (*n* = 37) available PPRV full‐length genomes (Figure 2). As expected from the sequence homology, the Bangladesh samples grouped strongly with the Indian and Tibetan isolates. The estimated time of divergence of this clade of viruses from other circulating lineage IV viruses is median TMRCA = 1998 (95% HPD 1978–1989).

**Figure 3:**
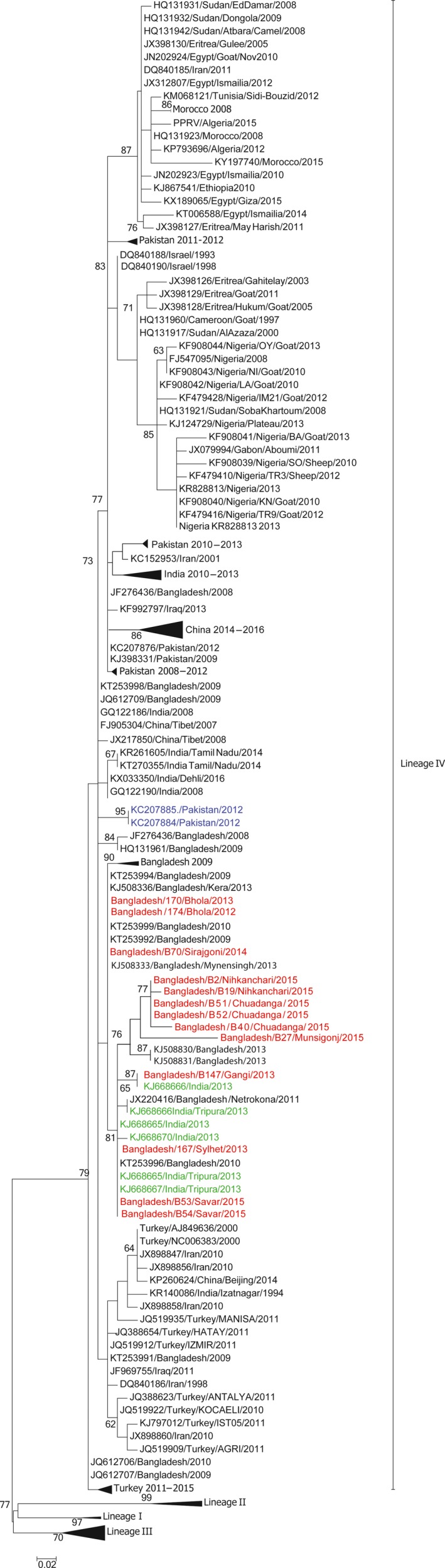
Neighbourhood‐joining tree using partial N gene sequences. Accession number, country of origin and sampling year of each isolate is shown. All sequences generated in this study are highlighted in red and isolates from the surrounding transboundary region of India are highlighted in green, and closely associated isolates from Pakistan highlighted in blue. All sequences generated in this study have been submitted to NCBI and awaiting accession numbers

As there are so few full‐length sequences available from the local region, a further 11 partial N sequences (255 nt) from Bangladesh collected between 2012 and 2015 were sequenced (Table 2). The partial N gene sequence of Bangladesh/B18/Nihkanchari/2015 was identical to that of Bangladesh/B19/Nihkanchari/2015; therefore was excluded from the analysis. These sequences (*n* = 10) were added to the equivalent regions extracted from the Bangladesh full‐length sequences (*n* = 3) (total sequences from this analysis *n* = 13) and 331 global partial N sequences of which almost half were from the surrounding region (Bangladesh *n* = 51, India *n* = 42, Pakistan *n* = 27, China including Tibet *n* = 30). Partial N sequences were extracted (as on 15/12/2017) from the GenBank repository (Supporting information Table S2) representing the available partial N sequences in GenBank for which accurate annotation details are available, and phylogenetic tree generated and annotated with bootstrap values (Figure 3). As has been observed previously (Munir et al., 2012; Muthuchelvan et al., 2014), the Bangladesh viruses cluster most closely with viruses from India, China, Tibet, Pakistan, and Iran. In particular, there are extremely strong relationships between viruses from the Indian border region of Tripura and from the Narayanganj and Netrokona outbreaks in Bangladesh, which have been previously sequenced (Muthuchelvan et al., 2014), as well as the isolates sequenced in this study. It is interesting that of the available Pakistani isolates (*n* = 27) two virus isolates both isolated from camels from the 2012 outbreak also grouped very strongly with virus isolates from Bangladesh (Figure 3). As Pakistan and Bangladesh do not share a border this further emphasizes the importance of the establishment of an effective regional approach to PPR eradication. This is of particular concern due to the porous nature of the border between India and Bangladesh to prevent the reoccurring transmission of PPR both between these nations but also further afield.

To conclude, this work describes the molecular detection of PPRV genome as well as isolation of virus from noninvasive samples (goat milk) collected from PPR outbreaks in Bangladesh. While there is currently no evidence for the direct transmission of PPRV through milk, it seems a likely pathway of vertical transmission of PPRV to kids and may be an additional factor in the high prevalence of PPRV mortality among kids (Taylor, 1984). Further investigations are required as to the possible transmission of PPRV between animals from goat milk. In particular, the length which virus remains present in milk and the effect of pasteurization on PPRV viability, as this data will have important implications for the development of effective controls for the export of milk products from PPR endemic regions as well as the development of testing methods for bulk milk storage. In additional, we have sequenced the full‐length viral genome of PPRV from milk and tissue samples from three isolates as well as the partial N gene sequence from a further ten isolates and used Bayesian phylogeography to demonstrate the transboundary nature of PPRV infection in the Indian subcontinent and further afield. The close relationships between viruses from Pakistan and Bangladesh serve in particular to emphasize the transboundary nature of PPRV as these countries do not share an immediate border, and highlight the importance of regional approaches to PPR control and eradication.

## CONFLICT OF INTEREST

The authors declare no conflict of interest.
