# Supplementary material for: Molecular detection, isolation and characterization of Peste‐des‐petits ruminants virus from goat milk from outbreaks in Bangladesh and its implication for eradication strategy
Source: Transbound Emerg Dis. 2018 May 28;65(6):1597–604. doi: 10.1111/tbed.12911 (PMC6282541; doi:10.1111/tbed.12911)
Supplement: Supplementary file 2 [file TBED-65-1597-s002.docx]

| **Supplementary Table 2** |
| --- |
| KX189065/Egypt/Giza/2015 |
| KT006588/Egypt/Ismailia/2014 |
| JX312807/Egypt/Ismailia/2012 |
| JN202923/Egypt/Ismailia/2010 |
| KC534492/Iran/2001 |
| KC152953/Iran/2001 |
| JX898847/Iran/2010 |
| JX898856/Iran/2010 |
| JX898858/Iran/2010 |
| JX898860/Iran/2010 |
| DQ840185/Iran/2011 |
| DQ840186/Iran/1998 |
| DQ840173/Israel/1993 |
| DQ840181/Israel/1993 |
| DQ840188/Israel/1993 |
| DQ840189/Israel/1993 |
| DQ840190/Israel/1998 |
| KF992797/Iraq/2013 |
| JF969755/Iraq/2011 |
| KR828813/Nigeria/2013 |
| KC594074/Morocco/2008 |
| KJ867541/Ethiopia2010 |
| KM089831/China/Henan/2014 |
| FJ905304/China/Tibet/2007 |
| JX217850/China/Tibet/2008 |
| KX033350/India/Dehli/2016 |
| KR261605/India/Tamil_Nadu/2014 |
| KT270355/India_Tamil/Nadu/2014 |
| KP260624/China/Beijing/2014 |
| KR140086/India/Izatnagar/1994 |
| Turkey/AJ849636/2000 |
| Turkey/NC006383/2000 |
| KU987612/Turkey/NIGDE/2015 |
| KU987610/Turkey/AKSARAY/2015 |
| KU325492/Turkey/ANTALYA/2015 |
| KU325487/Turkey/ANTALYA/2015 |
| KJ797012/Turkey/IST05/2011 |
| JQ519908/Turkey/ADANA/2011 |
| JQ519909/Turkey/AGRI/2011 |
| JQ519912/Turkey/IZMIR/2011 |
| JQ519916/Turkey/KAHRAMANMARAS/2011 |
| JQ519922/Turkey/KOCAELI/2010 |
| JQ519935/Turkey/MANISA/2011 |
| JQ519957/Turkey/SIVAS/2011 |
| JQ388623/Turkey/ANTALYA/2011 |
| JQ388654/Turkey/HATAY/2011 |
| JQ388664/Turkey/KAHRAMANMARAS/2011 |
| GQ122190/India/2008 |
| GQ122186/India/2008 |
| KT120058/India/2008 |
| KT120059/India/2008 |
| KM105872/India/2011 |
| KJ668665/India/2013 |
| KJ668666/India/2013 |
| KJ668670/India/2013 |
| KC200262/India/2012 |
| JN647694/India/1995 |
| JN632530/India/2007partial/cds |
| JN632532/India/2007 |
| DQ840177/India/1995 |
| KT253989/Bangladesh/2009 |
| KT253990/Bangladesh/2009 |
| KT253991/Bangladesh/2009 |
| KT253992/Bangladesh/2009 |
| KT253993/Bangladesh/2009 |
| KT253994/Bangladesh/2009 |
| KT253996/Bangladesh/2010 |
| KT253997/Bangladesh/2010 |
| KT253998/Bangladesh/2009 |
| KT253999/Bangladesh/2010 |
| JX220412/Bangladesh/2012 |
| JQ612706/Bangladesh/2010 |
| JQ612707/Bangladesh/2009 |
| JQ612709/Bangladesh/2009 |
| JF276436/Bangladesh/2008 |
| HQ131961/Bangladesh/2009 |
| PPRV/Algeria/2015 |
| KF908044/Nigeria/OY/Goat/2013 |
| KF908043/Nigeria/NI/Goat/2010 |
| KF908042/Nigeria/LA/Goat/2010 |
| KF908041/Nigeria/BA/Goat/2013 |
| KF908040/Nigeria/KN/Goat/2010 |
| KF908039/Nigeria/SO/Sheep/2010 |
| KF479428/Nigeria/IM21/Goat/2012 |
| KF479416/Nigeria/TR9/Goat/2012 |
| KF479410/Nigeria/TR3/Sheep/2012 |
| KF479408/Nigeria/TR1/Goat/2012 |
| KF483659/Mauritania/5/Sheep/2012 |
| JN202924/Egypt/Goat/Nov2010 |
| KC594074/Morocco/Goat/2008 |
| HQ131960/Cameroon/Goat/1997 |
| JX398126/Eritrea/Gahitelay/2003 |
| JX398127/Eritrea/May_Harish/2011 |
| JX398129/Eritrea/Goat/2011 |
| JX398128/Eritrea/Hukum/Goat/2005 |
| JX398130/Eritrea/Gulee/2005 |
| JX079994/Gabon/Aboumi/2011 |
| HQ131923/Morocco/2008 |
| FJ547095/Nigeria/2008 |
| HQ131917/Sudan/AlAzaza/2000 |
| HQ131942/Sudan/Atbara/Camel/2008 |
| HQ131922/Sudan/AbudelaiqKSUD/2008 |
| HQ131931/Sudan/EdDamar/2008 |
| HQ131921/Sudan/SobaKhartoum/2008 |
| HQ131945/Sudan/Rabak/2009 |
| HQ131932/Sudan/Dongola/2009 |
| KJ124729/Nigeria/Plateau/2013 |
| KP793696/Algeria/2012 |
| KM068121/Tunisia/Sidi-Bouzid/2012 |
| KF672746/Tanzania/Goat/2011 |
| KT692540/Benin/2011 |
| KP319027/China/2014 |
| Oman_KJ867544_1983 |
| UAE_KJ867545_1986 |
| Uganda_KJ867543_2012 |
| Kenya_KM463083_2011 |
| Ethiopia_KJ867540_1994 |
| Ivory_Coast_EU267273_1989 |
| Senegal_KP789375_1969 |
| Nigeria_KR828814_2012 |
| Benin_KR781449_2011 |
| Senegal_KM212177_2015 |
| Liberia_KU236379_2015 |
| Ivory_Coast_KR781451_2009 |
| Nigeria_EU267274_1976 |
| Benin_KR781450_1969 |
| Nigeria_KR828813_2013 |
| Morocco_KC594074_2008 |
| KY197740/Morocco/2015 |
| KJ668665/India/Tripura/2013 |
| KJ668666India/Tripura/2013 |
| KJ668667/India/Tripura/2013 |
| KT253998/Bangladesh/Din/FS9/2009 |
| KT253999/Bangladesh/2010 |
| KT253999/Bangladesh/Din/FS6/2010 |
| KJ398314/Pakistan/2012 |
| KJ398315/Pakistan/2012 |
| KJ398317/Pakistan/2012 |
| KJ398318/Pakistan/2012 |
| KJ398319/Pakistan/2012 |
| KJ398331/Pakistan/2009 |
| KJ398332/Pakistan/2010 |
| KJ398333/Pakistan/2010 |
| KJ398334/Pakistan/2010 |
| KJ398336/Pakistan/2009 |
| KJ398337/Pakistan/2008 |
| KJ398338/Pakistan/2008 |
| KJ398339/Pakistan/2009 |
| KJ398340/Pakistan/2009 |
| KC191631/Pakistan/2011 |
| KC191632/Pakistan/2011 |
| KC191633/Pakistan/2011 |
| KC191637/Pakistan/2011 |
| KC249964/Pakistan/2012 |
| KC207867/Pakistan/2012 |
| KC207875/Pakistan/2012 |
| KC207876/Pakistan/2012 |
| KC207885./Pakistan/2012 |
| KC207884/Pakistan/2012 |
| JN009673/Pakistan/2010 |
| KJ508829/Bangladesh/2013 |
| KJ508829/Bangladesh/Kera/2013 |
| KJ508830/Bangladesh/2013 |
| KJ508830/Bangladesh/Kera/2013 |
| KJ508831/Bangladesh/2013 |
| KJ508831/Bangladesh/Mymensingh/2009 |
| KJ508832/Bangladesh/2013 |
| KJ508832/Bangladesh/Mymensingh/2009 |
| KJ508833/Bangladesh/2013 |
| KJ508833/Bangladesh/Mymensingh/2013 |
| JN009674/Pakistan/2010 |
| Bangladesh/B170/Bhola/2012 |
| Bangladesh/B167/Sylhet/2013 |
| Bangladesh/B174/Bhola/2013 |
| Bangladesh/B147/Gangi/2013 |
| Bangladesh/B70/Sirajgonj/2014 |
| Bangladesh/B53/Savar /2015 |
| Bangladesh/B54/Savar /2015 |
| Bangladesh/B51/Chuadanga/2015 |
| Bangladesh/B52/Chuadanga/2015 |
| Bangladesh/B27/Munsigonj/2015 |
| Bangladesh/B2/Nihkanchari/2015 |
| Bangladesh/B19/Nihkanchari/2015 |
| Bangladesh/B40/Chuadanga/2015 |
